# “Low platelet usage” haematology laboratories: To store or not to store?

**DOI:** 10.1371/journal.pone.0187340

**Published:** 2017-11-13

**Authors:** M. Elmi, B. Sirigireddy, J. Abukar, D. Tchipeva, N. Chauhan, D. A. Tsitsikas

**Affiliations:** Haematology & Blood Transfusion Department, Homerton University Hospital NHS Foundation Trust, London, United Kingdom; FDA, UNITED STATES

## Abstract

In the UK, hospitals with annual platelet use of less than 500 pools, like our institution, usually do not store platelets on-site and these are only ordered from the national blood service (NBS) when a transfusion is required. In 2016, we piloted routine on-site storage of one pool of A RhD negative PLT. Data were collected retrospectively on units of PLTs ordered from NBS, units transfused to patients, wastage, requirements for emergency deliveries from NBS and overall cost. These were compared to corresponding data from the four preceding years (2012–2015). There was a 39% reduction in the PLT ordered from NBS in 2016 compared to previous years and a 50% reduction in transfused PLT. Annual wastage for 2016 increased by 23% even though the absolute number of wasted PLT did not alter significantly. Annual cost reduced by 36% in 2016 resulting from reduction in the total amount of PLTs ordered as well as reduction in emergency deliveries.

## Introduction

Platelet (PLT) transfusions are used to treat or prevent haemorrhage in at-risk patients [1]. However, they are a relatively scarce and costly resource with limited shelf life. In the UK platelet stocks are kept in hospitals with high demand such as those that have trauma centres, serve oncology patients or hospitals with specialised cardiac surgery services. In Trusts with “low PLT demands” (<400–500 pools /year) such as our own institution most often PLT are not kept on-site and only ordered from the national blood service (NBS) when a transfusion is required [2].

The demand for PLTs has increased by 25% in England from 2007 to 2015 [3] and our institution has also seen an increase of more than 30% in platelet transfusions from 2013 to 2014. This increase in platelet transfusions demand also restricts the NBS’s ability to stock healthy levels of PLTs.

Because of an increase in platelet transfusions and ad-hoc costs as well as the concerns around the safety of ordering PLT only when required, which often takes 2 hours to be delivered, in 2016 we piloted routine on-site storage of one pool of A RhD negative PLT and collected data on usage, wastage, cost and requirements for emergency orders from NBS.

## Methods

Data were collected retrospectively from the NBS customer service and from our Path manager system on a) pools of PLTs ordered from NBS, b) pools of PLTs transfused to patients, c) wastage, d) requirements for emergency deliveries from NBS and d) overall cost. These figures were compared to corresponding data from the four preceding years (2012–2015). As this was a retrospective data analysis of standard clinical practice, no prior power analysis for sample size calculation was carried out.

The cost of all platelets was valued at £193.15. Less than 1% of all platelets were either CMV negative or irradiated and came at a higher cost and were therefore not included in our analysis. The cost of each emergency delivery of PLT was £150.

To assess clinicians’ satisfaction, an online questionnaire was circulated via email to the 21 lead consultants in anaesthetics / Intensive Care, obstetrics and general surgery who are the main users in our institution. No further reminders were sent. This was an “in-house” generated questionnaire, not formally validated, aimed to provide us with a snapshot opinion of the new practice.

## Results and discussion

**Fig 1:**
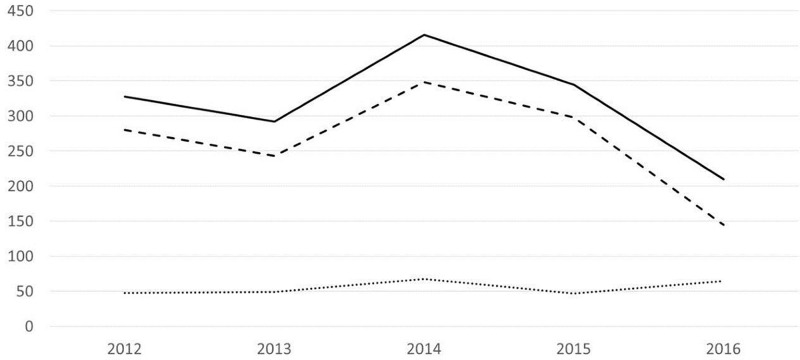
Platelet usage and wastage 2012–2016. Solid line = PLT ordered, dashed line = PLT transfused, dotted line = PLT wasted.

210 pools of PLT were ordered from NBS in 2016; this represents a 39% reduction from the annual order for the period 2012–2015 when the mean order of PLT/year was 345 pools (292–416). The pools of PLT transfused to patients was 145 in 2016, 50% less than 2012–2015 when the mean number of transfused PLT/year was 292 pools (243–348). Consequently, annual wastage increased: 65 pools of PLT were wasted in 2016 compared to an annual mean of 53 (47–68) between 2012–2015, representing a 23% increase. (Fig 1).

**Fig 2:**
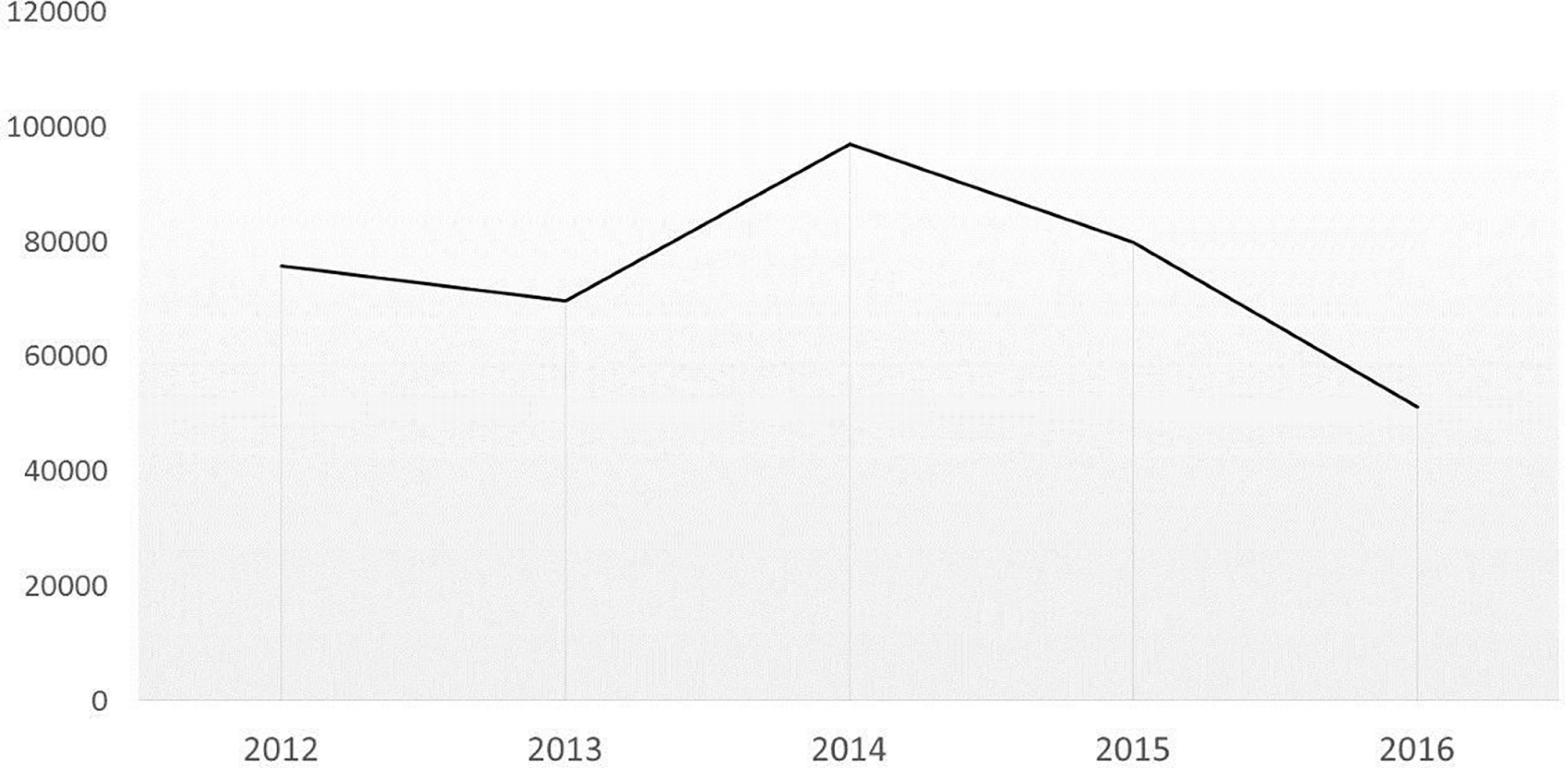
Overall cost (£) of PLT 2012–2016.

As anticipated, the annual cost for PLT orders from NBS also reduced by 39%: £40,562 in 2016 vs a mean of £66,685/year (£56,400 - £80,350) in 2012–2015. The annual number of emergency deliveries reduced by 23%: 71 in 2016 vs a mean of 92/year (82–110) in 2012–2015 with an associated cost of £10,586 in 2016 vs a mean of £13,819/year (£12,359 - £16,531) in 2012–2015. As a result, the overall cost for PLT usage to our service reduced by 36%: £51,148 in 2016 vs a mean of £80,504/year (£68,759 - £96,881) in 2012–2015 (Fig 2).

The clinicians’ satisfaction questionnaire yielded 16 responses. 13/16 (81%) agreed (3) or strongly agreed (10) that “Onsite storage of one pool of PLT increases patient safety” while 1/16 (6%) disagreed and 2/16 (13%) did not answer. 12/16 (75%) agreed (3) or strongly agreed (9) that “Keeping PLT on site encourages the clinicians to only request when certain of its usage” while 3/16 (19%) disagreed (2) or strongly disagreed (1) and 1/16 (6%) did not answer “S1 Table”.

The observed reduction in the pools of PLT transfused and the total amount of PLT requested from NBS cannot be explained on clinical grounds as the clinical services remained the same in the studied period while the total number of annual encounters gradually increased from 60,130 in 2012 to 70,458 in 2016 “Table 1”. At the same time, no other patient blood management (PBM) activities were undertaken.

**Table 1:** Annual total hospital encounters, platelets ordered from the national blood service and platelets transfused to patients.

Year	Discharges	PLT ordered (per encounter)	PLT transfused (per encounter)
2012	60130	328 (0.005)	280 (0.005)
2013	63783	292 (0.005)	243 (0.004)
2014	63698	416 (0.007)	348 (0.005)
2015	66656	345 (0.005)	298 (0.004)
2016	70458	210 (0.003)	145 (0.002)

These findings may suggest that previously, as PLT were not readily available, they may have been requested at the first suspicion they may become necessary and subsequently transfused even if not any more clinically indicated. Such practice may have been augmented by the short expiry date of PLT and a pressure to avoid wastage.

No PLT transfusion reactions were reported throughout the studied period (2012–2016).

This is a single-centre retrospective analysis on PLT usage, wastage and cost when a “low use” service stocks routinely one pool of PLT. We can only present collective data on PLT usage but cannot comment on individual transfusions and cannot therefore comment on the fluctuations observed in PLT usage in years 2012–2015. Nevertheless, we observe a reduction in the cost associated to emergency PLT deliveries during the studied period. Even though a direct link to our intervention cannot be proven, the use of PLT in 2016 was less than any individual year in the control period. A significant downside of this approach is an increased demand on A Rh negative PLT and a reduction in the use of group-specific PLT. However, it leads to significant cost savings while there is a suggestion it may also lead to a reduced demand of PLTs by the NBS. PLT wastage has increased significantly. This observation combined with the drastic reduction in the amount of PLT ordered and transfused, may also suggest that the increase in wastage mostly reflects reduction in inappropriate and unnecessary PLT transfusion.

## Conclusion

On-site PLT storage is associated with a cost benefit while there is a suggestion it may also lead to less PLT ordered and transfused. Other hospitals of similar size / PLT demand may benefit from a similar approach.

## Supporting information

S1 Table (DOCX)
