# Supplementary material for: “Low platelet usage” haematology laboratories: To store or not to store?
Source: PLoS One. 2017 Nov 13;12(11):e0187340. doi: 10.1371/journal.pone.0187340 (PMC5683636; doi:10.1371/journal.pone.0187340)
Supplement: S1 Table — (DOCX) [file pone.0187340.s001.docx]

|  | **Strongly Agree** | **Agree** | **Disagree** | **Strongly disagree** | **N/A** |
| --- | --- | --- | --- | --- | --- |
| Overall BT laboratory service meets our clinical needs for platelet transfusion requirements. | 4 | 9 | 1 | 0 | 2 |
| Onsite storage of a pooled platelet unit increases patient safety | 10 | 3 | 1 | 0 | 2 |
| Keeping PLT on site encourages the clinicians to only request when certain of its usage | 9 | 3 | 2 | 1 | 1 |
| Present PLTs turnaround time is adequate for my clinical needs | 4 | 9 | 1 | 0 | 2 |
